# “We don't need services. We have no problems”: exploring the experiences of young people who inject drugs in accessing harm reduction services

**DOI:** 10.7448/IAS.18.2.19442

**Published:** 2015-02-26

**Authors:** Anita Krug, Mikaela Hildebrand, Nina Sun

**Affiliations:** 1 Youth RISE, London, UK; 2 UNAIDS, Geneva, Switzerland

**Keywords:** young people, adolescents, drugs, injecting drug use, harm reduction, HIV

## Abstract

**Introduction:**

Evidence suggests that people who inject drugs often begin their drug use and injecting practices in adolescence, yet there are limited data available on the HIV epidemic and the responses for this population. The comprehensive package of interventions for the prevention, treatment and care of HIV infection among people who inject drugs first laid out in 2009 (revised in 2012) by World Health Organization, United Nations Office of Drugs and Crime and Joint United Nations Programme on HIV/AIDS, does not consider the unique needs of adolescent and young people. In order to better understand the values and preferences of young people who inject drugs in accessing harm reduction services and support, we undertook a series of community consultations with young people with experience of injecting drugs during adolescence.

**Methods:**

Community consultations (4–14 persons) were held in 14 countries. Participants were recruited using a combined criterion and maximum variation sampling strategy. Data were analyzed using collaborative qualitative data analysis. Frequency analysis of themes was conducted.

**Results:**

Nineteen community consultations were organized with a total of 132 participants. All participants had experienced injecting drugs before the age of 18. They had the following age distribution: 18–20 (37%), 21–25 (48%) and 26–30 (15%). Of the participants, 73.5% were male while 25.7% were female, with one transgender participant. Barriers to accessing the comprehensive package included: lack of information and knowledge of services, age restrictions on services, belief that services were not needed, fear of law enforcement, fear of stigma, lack of concern, high cost, lack of outreach, lack of knowledge of HCV/TB and lack of youth friendly services.

**Conclusions:**

The consultations provide a rare insight into the lived experiences of adolescents who inject drugs and highlight the dissonance between their reality and current policy and programmatic approaches. Findings suggest that harm reduction and HIV policies and programmes should adapt the comprehensive package to reach young people and explore linkages to other sectors such as education and employment to ensure they are fully supported and protected. Continued participation of the community of young people who inject drugs can help ensure policy and programmes respond to the social exclusion and denial of rights and prevent HIV infection among adolescents who inject drugs.

## Introduction

While the age distribution of the 12.7 million people who inject drugs globally is unknown [1], evidence suggests that people who inject drugs begin their injecting practices at a young age, often in adolescence [2]. Of the total population of people who inject drugs, 13.1%, or 1.7 million, were living with HIV in 2013 [1]. Globally, young people aged 15–24 years account for an estimated 35% of all new infections in people over 15 years of age [3]; yet data on the epidemic and response among young people who inject drugs (YPWID) are limited.

However, the data that do exist paints a stark picture. A number of countries have reported increases in prevalence of injecting drug use among young people [4] and high rates of HIV amongst adolescents who inject drugs [5,6]. YPWID are especially vulnerable to HIV [7,8]. Young people are more likely to share non-sterile injecting equipment [9]. As young people are new to the injecting community, they are less likely to know safer injecting practices [10]. In addition, sexual risk-taking takes place amongst YPWID [11] further increasing HIV risk. Legal age restrictions on harm reduction services prevents young people from accessing these services [12] and punitive measures that criminalize drug use further discourage service use thereby increasing HIV risk [13,14].

The comprehensive package of harm reduction services [15] has been endorsed by the World Health Organization (WHO), United Nations Office of Drugs and Crime (UNODC) and the Joint United Nations Programme on HIV/AIDS (UNAIDS) and are critical for reducing drug-related harms amongst people who inject drugs. The comprehensive package includes: 1) needle and syringe programmes (NSPs); 2) opiate substitution therapy (OSTs); 3) HIV testing and counselling; 4) antiretroviral therapy; 5) prevention of sexually transmitted infections; 6) condom programmes for people who inject drugs and their sexual partners; 7) targeted information, education and communication for people who inject drugs and their sexual partners; 8) vaccination, diagnosis and treatment of viral hepatitis; and 9) prevention, diagnosis and treatment of tuberculosis. However, current guidelines do not consider the unique needs of adolescent and young people or expand on how they could be adapted to ensure this age group is reached with services.

Formative research and/or meaningful community engagement that explore experiences of the “target population” can help develop programmes and policies that are effective [16]. Yet the participation of YPWID in policy and programme development cycles is largely absent [13]. To inform the current effort of the UNAIDS Inter-Agency Working Group on Key Populations (IAWGKP) to develop technical briefs on YPWID, Youth RISE with support from UNAIDS, undertook community consultations with young people who have experience injecting drugs during adolescence (10–19). This report presents the findings and discusses the implications for the comprehensive package of harm reduction services.

## Methods

### Community consultations

Given the dearth of data related to adolescents and YPWID, community consultations were used to generate in-depth information for the technical brief. A community consultation “is designed to recognize and accommodate the relevant particularities of a given community for a specific project” [17].

Consultations were organized in 14 countries, selected for convenience while ensuring geographical/regional diversity. Consultations were organized in different settings. In some countries, two smaller groups were held on the street. In Nepal and Nigeria, separate male and female consultations were held. As a result, a total of 19 consultations were organized: Indonesia (2), Kenya, Kyrgyzstan (2), Lebanon, Mauritius, Mexico, Nepal (2), Nigeria (2), Portugal, Romania, Slovenia, Ukraine, United States (2) and Vietnam. All consultations were conducted between August 2013 and January 2014.

The policy environment for OST and NSPs in the 14 countries was mapped and crosschecked with the 2012 Global State of Harm Reduction report [18]. Age of consent laws were mapped through review of literature [19–21].

A standardized consultation toolkit (a semi-structured discussion guide, a facilitator's guide, ethics protocol, informed consent and demographic information form) was developed by Youth RISE and UNAIDS, together with the community consultation facilitators. The kit was sent out for wider review by experts within the harm reduction field. Questions focused on experiences in accessing the comprehensive package of harm reduction services and how to improve access.

The consultations were facilitated by local young Youth RISE members. Facilitators were selected based on their experience with YPWID. All took part in a project and methodology workshop.

### Participants

Participants were recruited using a combined criterion and maximum variation sampling strategy [22]; that is, the facilitators purposefully recruited diverse participants that met a set of inclusion criteria. The initial criteria were: 1) have experienced injecting under the age of 18, and 2) aged between 18 and 25 years. Participants under the age of 18 were excluded due to ethical considerations. Consultation facilitators identified youth who met the inclusion criteria through services and/or street recruitment. Younger participants were hesitant to take part; consequently the age range was extended to 30 years of age to enable recruitment. While participants included both current and former injectors (regular and less regular), all participants met the inclusion criteria “having experienced injecting under the age of 18.” The discussions explored the experiences of adolescents, but as all participants were over 18 this report refers to young people.

### Data collection and analysis

The consultations were audio recorded, transcribed and translated into English where necessary. Data were analyzed using collaborative qualitative analysis [23]. Facilitators completed a standardized reporting template and the project coordinator independently coded the data [23]. The project coordinator and facilitator analysis were compared, and a preliminary report reviewed by all facilitators to verify data interpretation and findings. Once validated, frequency analysis of themes was conducted [23]. There were a total of 19 complete transcripts. If a theme was present in a majority of the transcripts (10+), it was considered a “strong” theme. If a theme was identified in a third (6–9), it was considered moderate. Unique themes were identified three or less of the transcripts, but offered a unique perspective in relation to the comprehensive package of interventions.

### Ethical protocol

IRB approval for the consultations was not obtained; however, an ethical protocol was followed, including: informed consent and confidentiality to protect identity. UNAIDS in-country offices advised on steps needed to ensure safety of participants, including engagement with Governments where necessary.

## Results

The results of the policy mapping on age restrictions are presented in Table 1 and indicate at what age adolescents can access these services without parental consent in the 14 countries. At the time of consultations, NSPs and OST are available in all countries except for Nigeria and Kenya [18]. Whilst the remaining interventions in the comprehensive package are available in all countries, the coverage of these programmes varies significantly and may not be specifically targeted towards people who inject drugs.

**Table 1:** Age restrictions on harm reduction services and HIV testing in countries where consultations took place

Country	City/location	Age restrictions (NSP)	Age restrictions (OST)	Age restrictions (HIV testing)
Indonesia	Bandung; Medan	None	Yes (18)	None
Kenya	Nairobi	None	No services	None
Kyrgyzstan	Bishkek	None	None	Yes (16)
Lebanon	Beirut	None	Yes (18)	No information identified
Mauritius	Port Louis	None	Yes (18)	None
Mexico	Hermosillo	Yes (18)	Yes (18)	Yes (18)
Nepal	Kathmandu; Pokhara	None	Yes (18)	Yes (14)
Nigeria	Abuja	No services	No services	Yes (18)
Portugal	Porto	None	Yes (18)	Yes (16)
Romania	Bucharest	Yes (18)	Yes (18)	No information identified
Slovenia	Ljubljana	None	16 (methadone); 15 (Subutex)	Yes (15)
Ukraine	Rivne	Yes (14)	Yes (14)	Yes (14)
United States	San Francisco	Varies by state (none in California)	Yes (18)	No information identified
Vietnam	Hanoi	Yes (16)	Yes (18)	Yes (16)

### Consultations

There were 132 participants in the consultations: age: 18–20 (*n*=49 (37%)), 21–25 (*n*=63 (48%)), 26–30 (*n*=20 (15%)); male: (*n*=97 (73.5%)); female: (*n*=34 (25.7%)), and genderqueer: (*n*=1 (0.8%)).

### Age of initiation into injecting drugs and reasons for initiation of injecting

Participants reported initiation into injecting as commonly starting between ages 15 and 18, although age of initiation was reported as young as nine. A process of progressing from cannabis, snorting drugs to finally injecting was commonly described. In all consultations, the need to get a more intense high was reported as the primary reason why injecting was initiated.

Curiosity was reported in a majority of the consultations as influencing adolescents’ decision to inject. Reduced quality and potency of drugs, as well as economic efficiency, were also reported as important reasons. Another was peer-influence:

For me at the beginning I rejected injecting drugs because I saw the blood inside the syringes and I know that it is dangerous and it's also dirty, but after several times saying no, I started to use the needle because I am just curious. I start to inject after about three times my friend offered for me to inject. (young man, Indonesia)

In the US and Indonesia, participants cited rejection from society or family as a point where they turned to harder drugs and/or injecting. “The reason I started injecting was because I was angry. I was expelled from school and abandoned by my own family when they found out I was taking some drugs. So I thought why not go all the way” (young man, Indonesia).

The gratification experienced from injecting drugs played an important role in why a young person continues to inject after their initial experience.

### Experiences accessing the comprehensive package of harm reduction

Participants identified barriers to accessing the interventions contained in the comprehensive package of harm reduction. Table 2 summarizes the frequency analysis of themes in relation to barriers identified.

**Table 2:** Adolescent and young people's identified barriers to accessing the comprehensive package of harm reduction services

Intervention and identified barriers	Theme strength
**Needle and syringe programmes**	
Lack of knowledge of services	Strong
Belief that services are not needed	Strong
Fear of police	Moderate
Fear of exposure of drug use	Moderate
Limited hours of operation	Moderate
Lack of youth-friendly services	Moderate
Age restrictions/parental consent requirements	Unique
Requirement of identity card	Unique
One-for-one exchange policies	Unique
**Opiate substitution therapy**	
Age restrictions/parental consent requirements	Strong
Belief that services are not needed	Strong
Lack of knowledge of service	Moderate
Cost	Moderate
Negative perception of OST and its side effects	Moderate
Registration of people who use drugs and lack of confidentiality	Unique
**HIV testing and counseling**	
Lack of concern	Strong
Cost	Strong
Lack of knowledge of services	Moderate
Stigma and fear of result	Moderate
Un-friendly staff	Moderate
Age of consent and/or parental consent requirements	Unique
**Antiretroviral therapy**	
Cost	Moderate
Age restrictions/parental consent requirements	Unique
Low testing and knowledge of status	Unique
Retention in ART services	Unique
**Prevention of sexually transmitted infections and condom programmes for IDUs and their sexual partners**	
Lack of concern	Strong
Effect of drugs on decisions around safe sex	Strong
Distribution of low quality condoms	Unique
Conservative social climate	Unique
**Targeted information, education and communication for IDUs and their sexual partners**	
No information received in adolescence	Strong
Lack of outreach	Strong
**Prevention, diagnosis and treatment of viral hepatitis**	
Lack of knowledge of HCV	Strong
Lack of concern	Strong
Cost	Moderate
**Prevention, diagnosis and treatment of tuberculosis**	
Lack of knowledge of tuberculosis	Strong
Lack of concern	Strong

#### Structural barriers

One commonly cited reason why participants did not access NSPs, OST and HIV services were age restrictions and/or parental consent requirements. While Mauritius has no age restrictions on NSPs, lack of clarity in the law has led to rejection of adolescents from NSPs and a lack of awareness among adolescents that services should be available to them. In all consultations, age restrictions were raised as a barrier to adolescents accessing OST. In Kyrgyzstan and Mexico, age of consent to HIV testing was also cited as a barrier where positive results are only released in the presence of parents and/or guardian.

A third of consultations described fear of police harassment and arrest as a reason why young people prefer not to access NSPs. Fear of law enforcement was also an important barrier for purchasing syringes from pharmacies. Cost and distance to services was identified as a barrier to accessing OST, HIV testing, viral hepatitis testing, and ART, as was the need to travel to centralized locations.

#### Social barriers

Fear of being exposed as a person who uses drugs led to hesitance amongst young people to access NSPs, and a preference for obtaining injecting equipment from pharmacies, which were perceived as more discrete.

In Kyrgyzstan, concern was raised about the registration of methadone clients and the impact that has on a young person's life, “Nobody wants to start on methadone at 18 because they will register you at once [...], You will have no normal life after that, no driving license, and they will give data on you everywhere, at school, local police and to doctors” (young female, Kyrgyzstan).

The two female-only consultations held in Nepal and Nigeria, as well as the mixed-group consultations, provided insights into how the needs of females differ. Participants indicated that females start injecting at a similar age as males; however, young women are less likely to be in contact with services and are more concerned about their drug use being exposed. A number of participants explained how their male partners initiated them into injecting, while heavy reliance on their partners for injecting equipment meant that accessing services was not necessary.

#### Lack of youth-friendly services

The presence of older users at harm reduction services and their attitude towards younger users made young people uncomfortable. “I think that we should have other types of services to young people under 18 years old. Because the CAT [Government run drug service in Portugal] is a bit aggressive. Can you imagine going into a CAT, in the middle of all that junkies on methadone and shit, we think, ‘What is this??’ [laughs]. Like this, young people don't feel like going into a CAT if they are having problems!” (Male, Portugal).

Judgemental attitudes of staff members towards young people who access HIV testing and counselling was repeatedly raised as a deterrent. Whilst young people largely preferred accessing injecting equipment from pharmacies, participants also reported that pharmacists’ attitudes were negative and that they sometimes refused to sell syringes.

#### Lack of information and risk-perception

Lack of knowledge of services was an important barrier to accessing all services. An adolescent who is new to the injecting community was unlikely to know of NSPs. A lack of knowledge of OST was also reported, particularly in adolescence; some apprehension about OST also existed with participants hearing about issues such as loss of teeth and a more intense withdrawal from methadone than heroin.

In a majority of the consultations, the participants said they had no knowledge of HIV testing in their adolescence. It was also suggested that YPWID only become aware of HIV testing sites when they begin drug treatment. Low knowledge of viral hepatitis and tuberculosis testing sites was reported in a majority of the consultations.

Information received about HIV prevention and treatment, safe sex and safe drug use varied considerably across consultations from very good information (e.g. US and Portugal) to none at all (e.g. Nigeria). However, in the majority of consultations, it was reiterated that information was only received after risky behaviours had already taken place.

Concern about STIs, viral hepatitis and tuberculosis was low across consultations. Whilst condoms were generally accessible according to participants, in a majority of the consultations it was reported that concern for safe sex disappeared after using drugs. “If you are high you do not care” (young man, Romania). In a majority of consultations, participants said that they were not concerned enough to get tested for HIV, believing it was a problem for “older users.”

The belief that services are not needed emerged strongly from the consultations. Participants described enjoying their drug use and not experiencing many negative consequences yet, and thus did not feel it necessary to seek out services. In the Ukraine, a participant put it simply: “We don't need services. We have no problems.” Similarly, a young woman in Slovenia said, “But when you are under age you mostly don't want OST. Cos those are the years when things are still good even if you are already addicted.”

#### Beyond the comprehensive package: additional support needs

Participants expressed that they require support beyond the comprehensive package of harm reduction. The following interventions were suggested in a third or more of the consultations: safe injecting advice; vocational training, removal of stigma and policy barriers to employment; parental engagement and education; support for street-involved youth; improved school drug education with a greater focus on risk reduction; legalization of softer drug; and legal education.

Young users need to be taught a) how to use, b) how to use correctly, and c) how not to die. (young man, US)

## Discussion

The consultations show that a person who injects drugs in their adolescence differs from older persons who inject drugs, which put them at greater risk of harm. The implications of the findings from the community consultations for the comprehensive package of harm reduction services recommended by WHO [15] is now discussed.

Participants agreed that young people begin using other drugs before using and injecting opiates. In a number of the consultations, perceived changes in drug trends amongst young people were discussed, generally from heroin to legal highs and synthetic drugs including amphetamine-type-stimulants (ATS). This trend has been reported elsewhere [1,24]. Whilst these drugs are more often not injected, injecting of synthetic and stimulant drugs also occurs [25]. Use of stimulant drugs in the party scene is commonplace amongst many young people [26], a population often not in contact with traditional harm reduction services. ATS users rarely use harm reduction services as they do not see these services as relevant [27]. In developing services to address adolescents and young people, different outreach strategies will be needed to reach both non-injecting and non-opiate substance use.

Reaching young people *before* they start injecting is an opportunity to prevent initiation into injecting, and/or to provide support and education to inject safely if injection drug use is initiated. Where young people experienced rejection by family, school and society over using “softer” drugs and started injecting as a coping-strategy also shows the importance of a more supportive response to non-injecting substance use.

While teaching an adolescent how to inject drugs may raise serious ethical and legal considerations for many policy makers and service providers, the fact that safe injecting education was identified as a need by participants within the majority of the consultations deserves further exploration.

Given the social context of initiation, working through social networks to prevent initiation into injecting may be an effective approach [28]. Initiatives and research on effective models is limited however, and further work is needed.

Consistent with other research [29], consultations found that even when NSPs are available for minors without parental consent, young people in the consultations consistently preferred to access their injecting equipment from pharmacies or friends, making the risk of sharing syringes greater. If adolescents prefer to obtain their syringes from pharmacies, it may be important to ensure pharmacies are able to provide information and education, engage in behavior change and link to drug treatment programmes and HIV testing and treatment programmes.

The consultations suggested that young people may be uncomfortable accessing services that older injectors frequent. Services that include a range of services that are not drug-specific may be more effective in engaging and serving adolescents. Integration of harm reduction interventions into other services already in contact with at-risk youth may also be a good approach. Lack of knowledge of services was another recurring theme, suggesting that a need for specific outreach strategies in order to reach younger people is necessary.

Adolescence is a period of experimentation and for many this includes experimentation with drug use [2]. During the initial period of drug use, consultations showed that young people may not necessarily identify themselves as “drug users at risk and/or in need of services.” The participants in the consultations often stressed pleasure as a key motivator for using drugs, and during this period where they may not be experiencing too many adverse effects, they are unlikely to reach out for support.

This has implications for how to successfully establish initial contact and engagement into services. More creative methods are needed to engage a young person who does not see him/herself as a “drug user,” whilst re-orienting services to be responsive to people who engage in experimental and enjoyable drug use as opposed to being services only relevant for those who are experiencing difficulties and/or want to stop using drugs may also lead to more successful engagement of younger people.

Under current WHO guidelines, there are no recommended age restrictions on NSP or OST programmes, yet these pose clear barriers to accessing NSPs and OST for adolescents [30]. The comparative review of age restrictions and parental consent requirements in the countries where consultations were organized indicate arbitrary restrictions, and such restrictions were repeatedly mentioned as a reason why young people who participated in the consultations were unable to access services. Countries should consider harmonizing their age-restrictions to international guidelines.

Another important yet often overlooked issue that arose consistently across the consultations was the impact of drug use on unsafe sex practices amongst adolescents, which supports previous research [31]. Programmes that better address the connection between sexual health and drugs are needed, particularly for adolescents.

Young women who use drugs are more vulnerable to HIV due to a number of age- and gender-specific vulnerabilities to both injection and sexual transmission routes [32]. Consultations indicated that young women are less willing to access services, have less knowledge about services, HIV, Hepatitis and TB, and frequently share injecting equipment with their male partners [33]. Outreach strategies are especially important for young women as they may be more dependent on their partners for injecting equipment and are fearful of the greater stigma placed on them, thereby resisting accessing services. Comprehensive health services that also address their sexual and reproductive health needs are needed [34].

### Study limitations

While working through a network of community activists proved an invaluable asset in building trust, some facilitators noted difficulties in recruiting participants due to fear of exposure as a person who uses drugs. In addition, all participants were over 18 and were questioned about their experiences of injecting drugs under 18. Recall bias inherent in this approach and self-reporting introduces further respondent biases. Whilst all participants reported that they currently inject drugs or have done so in the past, there was no process to verify whether participants did inject under the age of 18. Given the highly sensitive topic of drug use, the group rather than individual consultation approach may have led to minority perspectives not being raised. To mitigate this, the facilitators were selected based on their closeness to the community and were trained.

## Conclusions

The findings presented in this paper provide a rare insight into the lived experiences of YPWID and the challenges they face in accessing harm reduction services. Interestingly, experiences were fairly consistent across the 14 different countries. For example, the fact that YPWID do not identify as a “drug user in need of services” may provide insights into why current approaches to outreach and service delivery may be failing. The consultations indicated that adolescents and young people require significant support beyond the comprehensive package of harm reduction, with clear linkages to other sectors such as social security, education and employment.

While the findings are not representative, they speak of the importance of conducting formative and action research together with young people who use drugs to understand context specific barriers, social norms within the community, and the dissonance between legal and policy environment and practice. In addition, the empowerment process from participatory approaches should be valued in its own right. While drug use among adolescents and young people is a sensitive topic, it is hoped that the lived experiences of young people themselves can engender more honest conversations on how to best address the reality of injecting drug use among young people to reduce risk and harm.
